# Triboelectric–Electromagnetic Hybrid Generator for Harvesting Blue Energy

**DOI:** 10.1007/s40820-018-0207-3

**Published:** 2018-05-29

**Authors:** Huiyun Shao, Ping Cheng, Ruixuan Chen, Lingjie Xie, Na Sun, Qingqing Shen, Xiaoping Chen, Qianqian Zhu, Yi Zhang, Yina Liu, Zhen Wen, Xuhui Sun

**Affiliations:** 1 Institute of Functional Nano and Soft Materials (FUNSOM), Jiangsu Key Laboratory for Carbon-Based Functional Materials and Devices, and Joint International Research Laboratory of Carbon-Based Functional Materials and Devices, Soochow University, Suzhou, 215123 People’s Republic of China; 2 Institute of Advanced Materials (IAM), Nanjing Tech University, Nanjing, 211816 People’s Republic of China; 3 Department of Mathematical Sciences, Xi’an Jiaotong-Liverpool University, Suzhou, 215123 People’s Republic of China

**Keywords:** Triboelectric nanogenerator, Electromagnetic generator, Hybrid generator, Water flow, Power source

## Abstract

Progress has been developed in harvesting low-frequency and irregular blue energy using a triboelectric–electromagnetic hybrid generator in recent years. However, the design of the high-efficiency, mechanically durable hybrid structure is still challenging. In this study, we report a fully packaged triboelectric–electromagnetic hybrid generator (TEHG), in which magnets were utilized as the trigger to drive contact–separation-mode triboelectric nanogenerators (CS-TENGs) and coupled with copper coils to operate rotary freestanding-mode electromagnetic generators (RF-EMGs). The magnet pairs that produce attraction were used to transfer the external mechanical energy to the CS-TENGs, and packaging of the CS-TENG part was achieved to protect it from the ambient environment. Under a rotatory speed of 100 rpm, the CS-TENGs enabled the TEHG to deliver an output voltage, current, and average power of 315.8 V, 44.6 μA, and ~ 90.7 μW, and the output of the RF-EMGs was 0.59 V, 1.78 mA, and 79.6 μW, respectively. The cylinder-like structure made the TEHG more easily driven by water flow and demonstrated to work as a practical power source to charge commercial capacitors. It can charge a 33 μF capacitor from 0 to 2.1 V in 84 s, and the stored energy in the capacitor can drive an electronic thermometer and form a self-powered water-temperature sensing system.

**Electronic supplementary material:**

The online version of this article (10.1007/s40820-018-0207-3) contains supplementary material, which is available to authorized users.

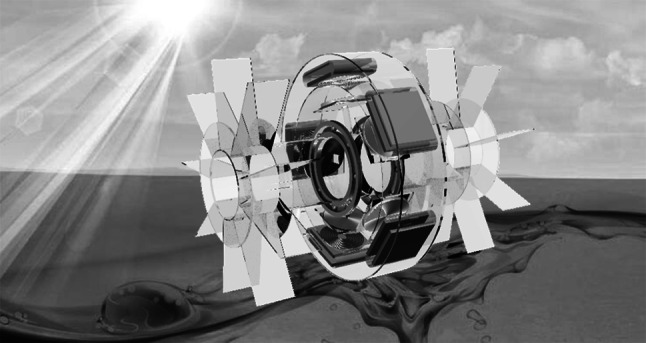

## Highlights

- A hybrid generator including contact–separation-mode triboelectric nanogenerators (CS-TENGs) and rotary freestanding-mode electromagnetic generators (RF-EMGs) with the potential to harvest water flow-based blue energy from the environment was designed.
- The magnet pairs that produce attraction were used to achieve packaging of the CS-TENGs part, protecting it from being affected by the ambient environment.
- In addition to powering light-emitting diodes, the generator can charge commercial capacitors and use the stored energy to power an electronic water thermometer.

## Introduction

Much effort has been made to meet the huge energy demand of modern society while minimizing environmental cost [1]. Widely distributed water kinetic energy is an abundant source for large-scale applications and is much less dependent on seasonality, day–night, weather, and temperature variations [2–4]. Especially in the form of water flow, it contains a gigantic reserve of kinetic energy, but is hardly utilized in an effective way [5–7]. Recently, triboelectric nanogenerators (TENGs) have emerged as a powerful technology for harvesting low-frequency mechanical energy with characteristics including lightweight, low cost, and wide selection of materials [8–13]. More improvements have been made in the use of TENGs to achieve a human–machine interface [14, 15]. Essentially, TENGs demonstrate much better output performance than that of traditional electromagnetic generators (EMGs) at low frequency (typically 0.1–3 Hz), which confirms the possible application of TENGs for harvesting irregular and low-frequency motion energy such as that from water flow [16, 17]. How to use this novel technology to achieve energy collection and conversion attracts much attention.

The original idea of using TENGs for water wave energy was proposed by the liquid–solid electrification-enabled process. During the submerging and surfacing process due to traveling water waves, current flows between the electrodes to screen the charges on the triboelectric layer of the TENG, thereby producing electric power [18–20]. However, the output performance decreases dramatically to almost zero at a high ion concentration in a real water environment owing to the streaming potential theory [21–23]. An additional strategy was put forward involving a freestanding, fully enclosed TENG that packs a rolling ball in its interior to form a rocking spherical shell [24–27]. Later on, various designs based on the hybridization of TENGs and EMGs were developed [28–31]. The magnet pairs of EMGs produce the noncontact attractive force that enables the fully enclosed packaging of the TENG part, protecting it from the ambient environment. Meanwhile, the complementary outputs can be hybridized and maximized in a broad frequency range. Nevertheless, these hybrid generators are still in the development stage for water flow energy collection, and more research is highly desired to optimize their structure and improve their performance toward practical applications.

In this work, we present the design of a hybrid generator based on contact–separation-mode TENGs (CS-TENGs) in conjunction with rotary freestanding-mode EMGs (RF-EMGs). Five CS-TENGs were initially fixed in an enclosed cylinder to isolate the impact of water. Relying on the attraction force between the magnets, two triboelectric layers of the CS-TENG contact and separate periodically during the rotation process. The device durability is greatly enhanced with respect to that of TENGs based on the sliding mode. This ingenious design combines the output feature of both CS-TENGs and RF-EMGs at different rotation speeds. Remarkably, compared with other structures, the cylinder-like structure is easier to be driven by water flow. Water flow impacts the impeller, allowing the device to rotate at a steady rate. Furthermore, the device was installed in a turbulent place to directly power LEDs and clearly demonstrated a higher output from the CS-TENGs at low frequency and from the RF-EMGs at high frequency. As a demo, it can also charge commercial capacitors and use the stored energy to power an electronic water thermometer.

## Experimental Section

### Fabrication of Nanowire Array on Polytetrafluoroethylene Surface

The nanowire array was created on a polytetrafluoroethylene (PTFE) surface by a one-step plasma reactive ion etching process reported previously. The PTFE films were cleaned with alcohol, isopropyl alcohol, and deionized water successively and then dried in an oven at 50 °C. A thin layer of Cu film was deposited on the cleaned PTFE surface by sputtering. Then, inductively coupled plasma (ICP) etching was utilized to produce aligned nanowire-like structures on the surface. Specifically, Ar, O_2_, and CF_4_ gases were added in the ICP chamber with flow ratios of 15.0, 10.0, and 30.0 sccm, respectively. A power of 400 W was used for plasma generation, and a power of 100 W was used for accelerating the plasma ions. The PTFE film was etched for 6 min to obtain the nanowire-like structures.

### Assembly of the Hybrid Generator

First, two acrylic sheets with a size of 40 × 40 mm^2^ were shaped by a laser cutter as the substrates and a thin Al foil (40 × 40 mm^2^) was then attached to the top substrate as the top electrode, and a thin Al foil of the same size was attached to the bottom substrate. The surface of the Al foil was covered by the PTFE film. The nanostructures were fabricated on the surface of the PTFE film by ICP etching. In an RF-EMG unit, a coil was inserted between two magnets. Two acrylic cylinders with the same width but different diameters were sheathed and fixed together on two acrylic disks to form a closed space. Five coils were arranged on the outer surface of the smaller acrylic cylinder at equal spacings. Next, a CS-TENG was fixed on each coil. Then, a magnet was fixed at the top of each CS-TENG. The closed space contained the CS-TENG part and the stator part of the RF-EMG. A thin acrylic tube passed through the center of the closed cavity and was connected to it via two bearings. Five magnets were equally spaced and arranged in the middle of the tube, and two impellers were distributed at both ends of the tube.

### Electrical Measurement

The surface morphology of the PTFE thin film was characterized by scanning electron microscopy (SEM, FEI Co., model Quanta-200). The output voltage signal and the output current signal were acquired via a programmable electrometer (Keithley model 6514). The software platform was constructed using LabVIEW and was capable of realizing real-time data collection and analysis. A rotating motor (86BYG250D, MA860H) was applied to drive the device to rotate.

## Results and Discussion

**Fig. 1:**
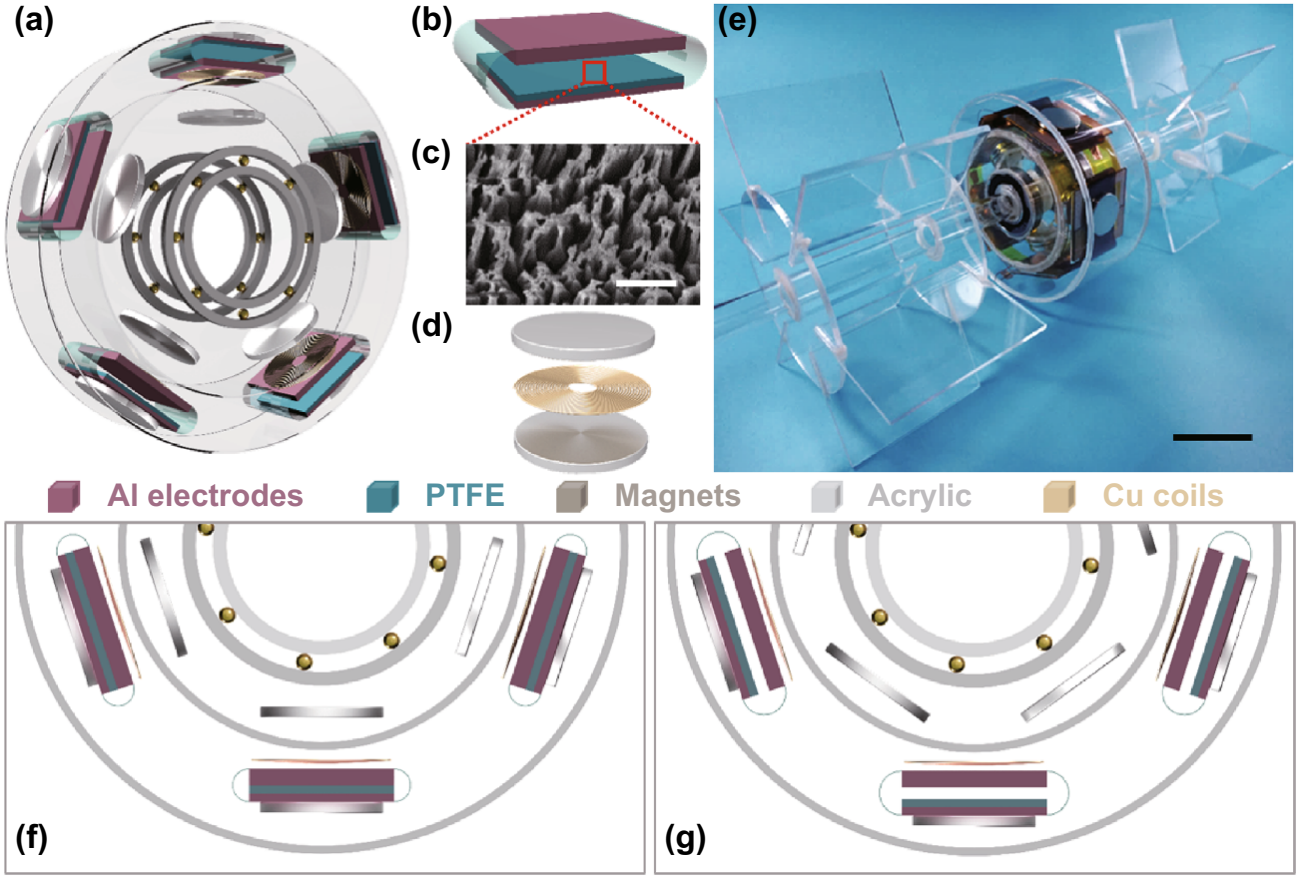
Structure of the triboelectric–electromagnetic hybrid generator (TEHG). **a** Schematic illustration of the as-fabricated TEHG, which consists of five contact–separation-mode triboelectric nanogenerators (CS-TENGs) and five rotary freestanding-mode electromagnetic generators (RF-EMGs). Detailed schematic illustration of **b** a CS-TENG and **d** a RF-EMG. **c** SEM image of the polymer nanowires on the PTFE film (scale bar: 1 μm). **e** Tilted-view photograph of an as-fabricated TEHG (scale bar: 3 cm). Cross-sectional view of the TEHG **f** when the rotary magnets are aligned with copper coils and **g** when the rotary magnets gradually move away

The proposed TEHG is a fully integrated device composed of five CS-TENGs and the corresponding RF-EMGs, as schematically illustrated in Fig. 1a. For typical CS-TENGs, two acrylic sheets were shaped by the laser cutter as the double-layered acrylic substrates. A thin Al foil was attached to the top acrylic substrate, and another thin Al foil with the same size was attached to the bottom substrate. After connecting the Cu wires with two electrodes, a PTFE film covered the surface of the bottom Al foil as a triboelectric layer and a CS-TENG was obtained (Fig. 1b). To enhance the electric output of the CS-TENG, nanowires with a diameter and length of ~ 100 and ~ 250 nm, respectively, were fabricated on the surface of the PTFE film by ICP etching, providing a large contact area to generate more triboelectric charges (Fig. 1c). For the RF-EMGs, a copper synclastic twined coil was placed between two magnets, forming a sandwich structure (Fig. 1d). The photograph of a typical as-fabricated device is shown in Fig. 1e. For convenience of demonstration, the framework of the whole unit was constructed using transparent acrylic materials. A pair of concentric cylinders was sheathed and fixed together on two disks to form a closed space that could hold five CS-TENGs. Five coils were arranged on the outer surface of the smaller acrylic cylinder at equal spacings. Then, a CS-TENG was fixed on each coil and a magnet was fixed on the top of each CS-TENG. The closed space contained the CS-TENG part and the stator part of the RF-EMG. A thin acrylic tube passed through the center of the closed cavity and was connected to it through two bearings. Five magnets were equally spaced and arranged in the middle of the tube, and two impellers were distributed at both ends of the tube. These rotary magnets worked as a rotary trigger for the CS-TENGs and as a rotator for the RF-EMGs. When the impellers were rotating, owing to the attractive force produced by the magnet pairs, the magnet pairs periodically approached (Fig. 1f) and separated (Fig. 1g), causing both the periodic trigger of the CS-TENGs and magnetic flux changes in the copper coils to produce the electrical output. The linkage mechanism of the triboelectric–electromagnetic hybrid generator is shown in Fig. S1. In virtue of the rotation of the two impellers under flowing water, water flow energy can be converted into electric energy.

**Fig. 2:**
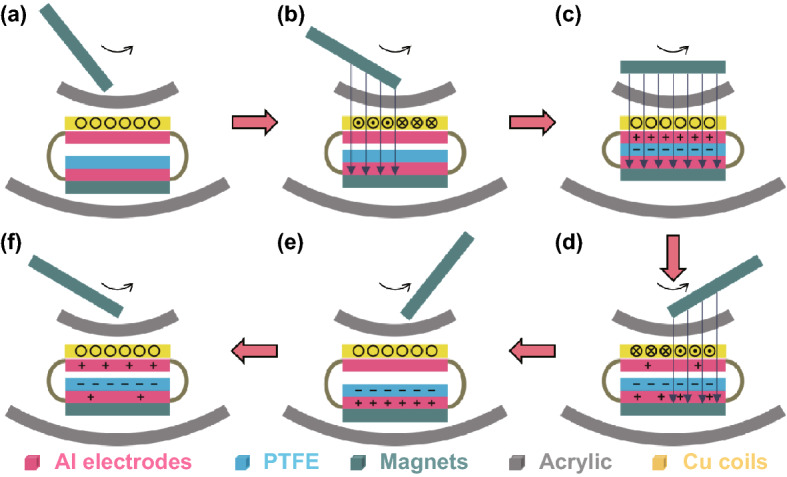
Schematics of the operating principle of the TEHG. **a** Initial state. **b** Current generation process of the RF-EMG. **c** The rotary magnet is aligned to copper coil. **d** The rotary magnet gradually moves away. **e** The CS-TENG completely recovers its shape. **f** Another magnet approaches the CS-TENG unit

The electric energy produced by the TEHG consists of two parts, one part from the CS-TENG and another from the RF-EMG, as schematically depicted in Fig. 2. Herein, two-dimensional schematic illustrations of the current and charge distribution of the CS-TENG and the magnetic flux of the RF-EMG are employed to elucidate the working mechanism of the minimum functional unit. The working mechanism of the CS-TENG, which can be referred to as a common contact–separation-mode TENG, is based on the coupling between contact electrification and electrostatic induction [32–36]. Under external triggering, such as the water flow impact, the impellers begin to rotate, driving the magnets to move together. Then, the PTFE film periodically contacts and separates from the Al foil. To simplify the description, we named the rotary magnet as the upper magnet and the stationary magnet as the bottom magnet. Initially, when the upper magnet is fully misaligned with the bottom magnet, the two magnets are far apart, resulting in a weak magnetic field between them (Fig. 2a). With further rotation of the upper magnet, the two surfaces are close to each other, but there is still no charge transfer (Fig. 2b). When the upper magnet is aligned with the bottom magnet, the attractive force between the two magnets is applied to the CS-TENG, which brings the PTFE film into contact with the Al foil, and charge transfer occurs at the contact interfaces. According to the static charge triboelectric series, PTFE is much more triboelectrically negative than Al, and thus, the electrons are injected from the Al into the PTFE, generating positive tribocharges on the Al and negative ones on the PTFE (Fig. 2c). If the upper magnet gradually moves away, the elasticity of the Kapton film will lead to a separation between the PTFE and the Al. Afterward, electrical potential difference is created between the two electrodes, resulting in an instantaneous current attributed to the electron flow from the bottom electrode to the top electrode (Fig. 2d). Then, the CS-TENG completely recovers its shape and the negative tribo-charges are almost totally neutralized by the inductive positive charges (Fig. 2e). When another magnet approaches the CS-TENG unit, the two triboelectric layers get close to each other again, and the transferred charges flow back to the surface of the top electrode, forming a reverse current (Fig. 2f). When the two surfaces are in full contact, as depicted in Fig. 2c, a cycle of the electricity generation process of the CS-TENG is completed. Obviously, the rotary magnet can not only trigger the CS-TENG through the change of the contact–separation state, but can also trigger the RF-EMG through the change of the magnetic flux in the coil. For the RF-EMG unit, it is based on Faraday’s law of electromagnetic induction [37–40]. When a permanent magnet moves from state **a** to state **b**, the magnetic flux crossing the copper coil will increase until it reaches a maximum. Similarly, the magnetic flux crossing the copper coil decreases from state c to state e. When the device rotates, the RF-EMG enables the delivery of an alternating current through a periodic change of the magnetic flux in the coil, which is monitored in the external circuit.

**Fig. 3:**
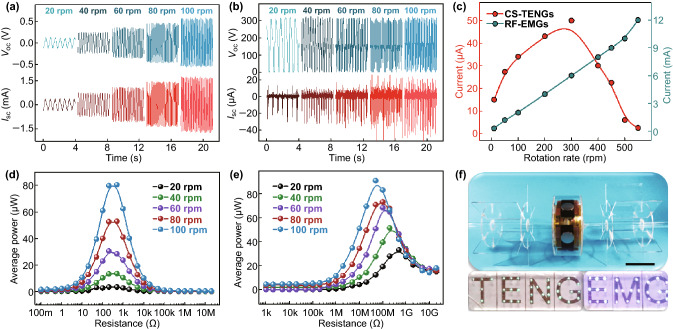
Electrical output performance of each functional component of the TEHG. *V*_oc_ and *I*_sc_ of **a** five RF-EMGs connected in series and **b** five CS-TENGs connected in parallel at a rotation speed ranging from 20 to 100 rpm. **c** Dependence of the output current on the rotation rate of the TEHG. Dependence of the average power of **d** the RF-EMGs and **e** the CS-TENGs on the external load resistances. **f** The words “TENG” and “EMG”, made up from LEDs, were lighted (scale bar: 5 cm)

The electrical output performances of the CS-TENG and RF-EMG, including open-circuit voltages (*V*_oc_), short-circuit currents (*I*_sc_), and average power, are shown in Fig. 3. A rotary motor that can produce rotating motion at a fixed speed was employed to drive the rotating shaft of the device. The measurements were taken using a speed from 20 to 100 rpm according to the inherently low and variable rate of water flow, which is very critical for practical application. The voltage and current output of five RF-EMGs connected in series at five speeds are plotted in Fig. 3a. It was found that the *V*_oc_ increases from ~ 0.15 to 0.59 V as the rotation speed increases, while the current also increases from ~ 0.39 to 1.78 mA. For five CS-TENGs connected in parallel, *V*_oc_ keeps a constant peak value of ~ 315.8 V, while *I*_sc_ increases proportionally from ~ 24.5 to ~ 44.6 μA, as displayed in Fig. 3b. The transferred charges (*Q*_sc_) of five parallel CS-TENGs under different rotation speeds are displayed in Fig. S2. Figure S3 shows that less than ± 10% of electrical output fluctuation was observed after continuous operation for 30,000 cycles (with a fixed rotation speed of 50 rpm for 10 h), demonstrating the robustness and stability of the device. Comparisons with a similar hybrid generator combining TENGs and EMGs for harvesting blue energy are listed in Table S1. The dependence of the output current of the TEHG on the rotation speed is shown in Fig. 3c. At first, the output current of the CS-TENGs increases rapidly with increasing rotation speed and then it is gradually saturated. Afterward, it begins to decline and finally decreases to almost zero. It is worth noting that when the rotation speed exceeds a large value, the two triboelectric layers cannot contact each other. However, the tendency of the current of the RF-EMGs is totally different. Initially, the current of the RF-EMGs remains stable at a low output stage and then rises rapidly. The results indicate that the different output trends play complementary roles to each other. The corresponding average power of the RF-EMGs is displayed in Fig. 3d. The average power is maximized at an external load of ~ 318 Ω for all rotation rates, and the corresponding maximum power is ~ 3.9, 13.8, 29.7, 52.6, and 79.6 μW. The optimized average power density of the RF-EMGs is proportional to the square of the rotation speed (~ 0.11 to ~ 2.25 μW cm^−2^). The low output is mainly attributed to the low speed of the magnets. It can be enhanced by increasing the number of coils or the strength and number of the magnets, or by improving the rotational speed. Figure 3e displays the average power of the CS-TENGs as a function of external load at five rotation speeds. Unlike the RF-EMGs, the external resistance that corresponds to the maximum power varies with the rotation speed. The maximum average power equals to 32.9, 51.2, 68.2, 73.1, and 90.7 μW. The optimized average power density of the CS-TENGs is proportional to the rotation speed (~ 0.41 to ~ 1.13 μW cm^−2^). However, the CS-TENGs have a lower output compared to some reported similar devices. The possible reason for this low output may be that the attraction between the magnets is not large enough, which may result in insufficient contact between the surfaces of the two dielectric materials. On the one hand, we can increase the number of magnets appropriately and shorten the distance between the magnets to increase the output. On the other hand, we can choose better dielectric materials for the TENGs. To demonstrate a practical application of the TEHG powering external loads, a high-pressure water gun was employed to simulate the water flow in the laboratory. By regulating the water pressure, the velocity of the water flow can be switched between high speed and low speed. To directly exhibit the results, we connected some green LEDs in series with the CS-TENGs and some blue LEDs in parallel with the RF-EMGs. Figure S4 shows the circuit diagram of the device with five CS-TENG units and five RF-EMG units to power the LEDs. A photograph of the experimental setup is shown in Fig. 3f. As the water flow passed by, the tremendous impact of the water acted as a power source to drive the device. When the water pressure was small, only the word “TENG” made up of LEDs was lit up. Because the voltage of the EMG cannot exceed the threshold voltage of the LEDs, the word “EMG” could not be lit up (Supporting Movie S1). Once the water pressure was large enough, the two components could supply power to the LEDs together (Supporting Movie S2). Based on the device configuration, we can effectively utilize the output of the two parts, whether the water flow is violent or gentle.

**Fig. 4:**
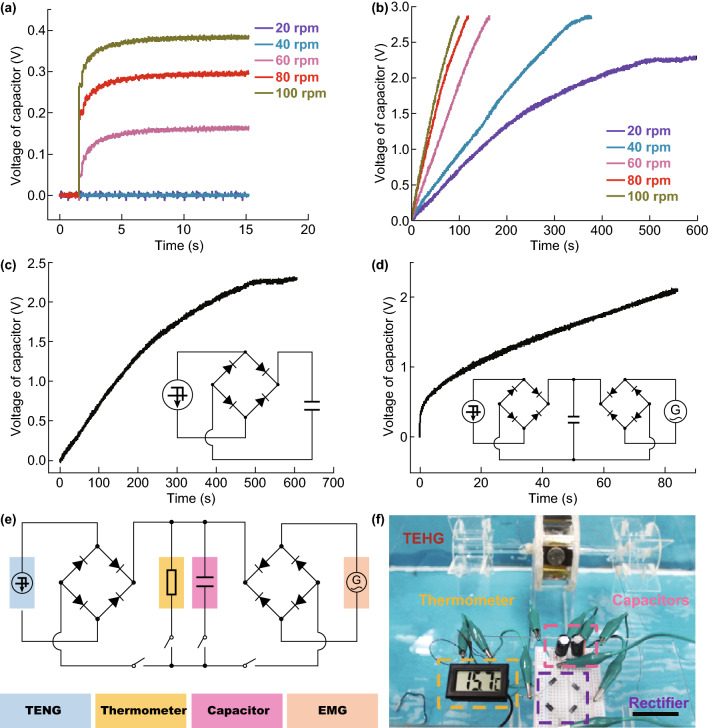
Distinctive and combined performances of the RF-EMG and CS-TENG. Measured voltages of a 33 μF capacitor charged by **a** the RF-EMGs and **b** the CS-TENGs at a rotation speed ranging from 20 to 100 rpm. Measured voltages of a 33 μF commercial capacitor charged by the TEHG under two different working conditions: **c** at 40 rpm and **d** at 100 rpm. The inset shows the circuit diagrams. **e** Circuit diagram of a self-powered water-temperature sensing system. **f** Photograph of the self-powered water-temperature sensing system, including the TEHG, rectifier, capacitors, and electronic thermometer (scale bar: 4 cm)

The efficient usage of the generated energy by the TEHG is distinctly different at different rotation speeds. To further evaluate and compare the overall energy output capability of the RF-EMGs and CS-TENGs, a series of experiments and demonstrations were performed, as shown in Fig. 4. The figure depicts the charging voltage curves of the commercial capacitor used for the CS-TENG, RF-EMG, and TEHG. With a full bridge rectifier, the voltage of a 33 μF capacitor can be charged by the RF-EMGs, which was measured at speeds ranging from 20 to 100 rpm, as shown in Fig. 4a. Theoretically, there should be a positive correlation between the speeds and the voltage of capacitor. However, at low speeds, such as 20 and 40 rpm, the voltage across the capacitor is 0. A ~ 0.2 V voltage drop is required to trigger a full-wave bridge rectifier. The saturation voltage of the RF-EMGs is limited by the low rotation speed. Consequently, the low voltage output produced by the RF-EMGs can hardly be stored inside a capacitor at a low rotation speed (< 40 rpm). At relatively high speeds, the capacitor enables us to obtain the voltage shown in the corresponding curves. For the CS-TENGs, the charging voltage as a function of the charging time was measured by a voltage meter and is shown in Fig. 4b. The CS-TENGs can charge a 33 μF capacitor to 2.87 V in about 99 s at a speed of 100 rpm. Recently, a designed charging cycle has been reported to achieve much more effective energy storage with improved charging rate and enhanced energy storage efficiency [41]. Alternatively, the charging rate can also be improved greatly through a power management circuit board [42]. In summary, the charging efficiency can be greatly enhanced through the designed charging cycle and a power management system. The dependence of the rectified current on the rotation speed of the CS-TENGs is presented in Fig. S5. For more efficient energy harvesting and storage, the two parts of the CS-TENGs and RF-EMGs can be combined. Additionally, an integrated system including the electricity generation, rectification, and storage was developed. The charging voltage curves of a capacitor using the TEHG are plotted in Fig. 4c, d. The figure shows that the charging characteristics heavily depend on the inherent output performance of the RF-EMG and CS-TENG. The output voltage determines the final charging level, whereas the output current determines the charging speed. We simulated two different working conditions and defined the working state under 40 rpm as the low-speed state. Only the output voltage of the CS-TENGs contributes to the voltage rise of the capacitor. Owing to the high output voltage and low output current of the CS-TENG, the capacitor can be charged up to the maximum open-circuit voltage with a long charging time. Similarly, operation at 100 rpm was regarded as the high-speed state. The voltage of the capacitor was rapidly saturated within a short charging time, and then the capacitor could be charged continuously by the CS-TENGs to a higher voltage. The voltages of the capacitors at both speed states rose to ~ 2.1 V, but the high speed required less time. As a result, the faster the device runs, the more efficient the energy harvesting and storage will be. A commercial water thermometer can be easily powered by the TEHG as a demo. The circuit diagram and a photograph of the working process of the self-powered water-temperature sensing system are shown in Fig. 4e, f. The temperature of the water can be recorded and visualized through a liquid crystal display until the thermometer automatically turns off, once the voltage of the capacitors is too low; then, the capacitors begin to recharge. The above energy supply process for a thermometer with specific power consumption requirements is very representative. Most sensors in unattended water-monitoring systems have an intermittent operating mode to collect or send data and require discontinuous high-power consumption. The above concept and design can offer a feasible power solution for long-term, wide-area, in situ, real-time monitoring of water parameters and be a favorable power choice, particularly in closed environments.

## Conclusion

In summary, we have designed and demonstrated a hybrid generator including CS-TENGs and RF-EMGs with the potential to harvest water flow-based blue energy from the environment. The output performance of the CS-TENGs and RF-EMGs was measured under the regular action of the rotary motor, and the key concept and design of our device are to combine the two generators together. Thus, the CS-TENG can harvest low-frequency energy, whereas the RF-EMG produces larger output in a high-frequency range. The generated output from the RF-EMGs can reach a peak voltage of 0.59 V and a peak current of 1.78 mA at 100 rpm. For the CS-TENGs, an output voltage and current of 315.8 V and 44.6 μA, respectively, were achieved at 100 rpm, demonstrating the applicability of the generator in a real environment. Moreover, although the CS-TENGs can directly drive a series of LEDs at low or high rates, the RF-EMGs can only light up all the LEDs at high rates. The magnet pairs that produce the attraction force are used to achieve packaging of the CS-TENG part, protecting it from the external environment. The rectified outputs have also been demonstrated to charge commercial capacitors, whose stored energy can power an electronic thermometer in a self-powered water-temperature sensing system.

## Electronic supplementary material

Below is the link to the electronic supplementary material.

Supplementary material 1 (PDF 419 kb)
