# Supplementary material for: Triboelectric–Electromagnetic Hybrid Generator for Harvesting Blue Energy
Source: Nanomicro Lett. 2018 May 29;10(3):54. doi: 10.1007/s40820-018-0207-3 (PMC6199107; doi:10.1007/s40820-018-0207-3)
Supplement: Supplementary file 1 — Supplementary material 1 (PDF 419 kb) [file 40820_2018_207_MOESM1_ESM.pdf]

Supporting Information for

Triboelectric–Electromagnetic Hybrid Generator for Harvesting Blue Energy

Huiyun Shao[1, #], Ping Cheng[1, #], Ruixuan Chen[1, 2, #], Lingjie Xie[1], Na Sun[1], Qingqing Shen[1], Xiaoping Chen[1], Qianqian Zhu[1], Yi Zhang[1], Yina Liu[3], Zhen Wen[1,*], Xuhui Sun[1,*]

[1]Institute of Functional Nano and Soft Materials (FUNSOM), Jiangsu Key Laboratory for Carbon-Based Functional Materials and Devices, and Joint International Research Laboratory of Carbon-Based Functional Materials and Devices, Soochow University, Suzhou 215123, People's Republic of China

[2]Institute of Advanced Materials (IAM), Nanjing Tech University, Nanjing 211816, People's Republic of China

[3]Department of Mathematical Sciences, Xi'an Jiaotong-Liverpool University, Suzhou 215123, People's Republic of China

[#] Huiyun Shao, Ping Cheng and Ruixuan Chen contributed equally to this work

*Corresponding authors. E-mail: wenzhen2011@suda.edu.cn (Zhen Wen); xhsun@suda.edu.cn (Xuhui Sun)

Supplementary Figures and Table

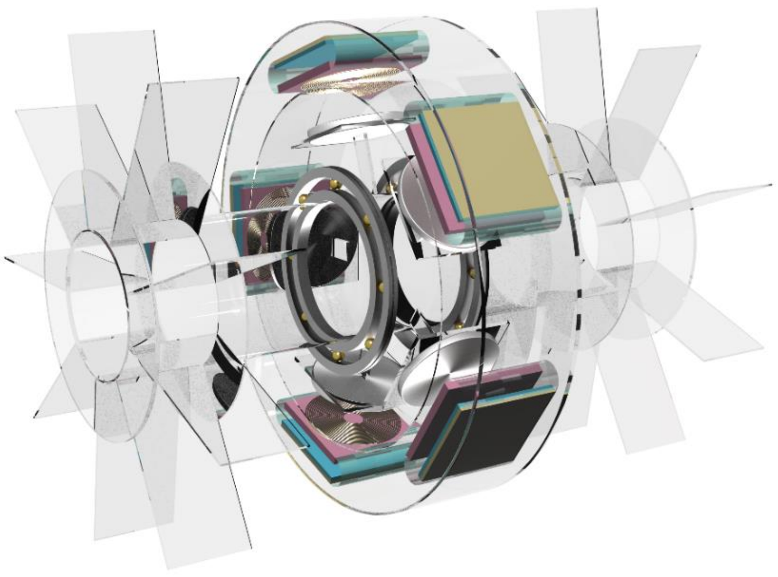

Fig. S1 The linkage mechanism of the triboelectric-electromagnetic hybrid generator

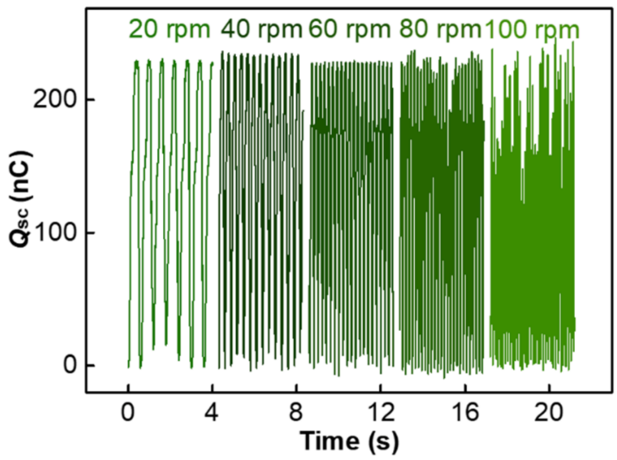

Fig. S2 The transferred charges (Q_{sc}) of five-parallel CS-TENGs under different rotation speed ranging from 20 to 100 rpm

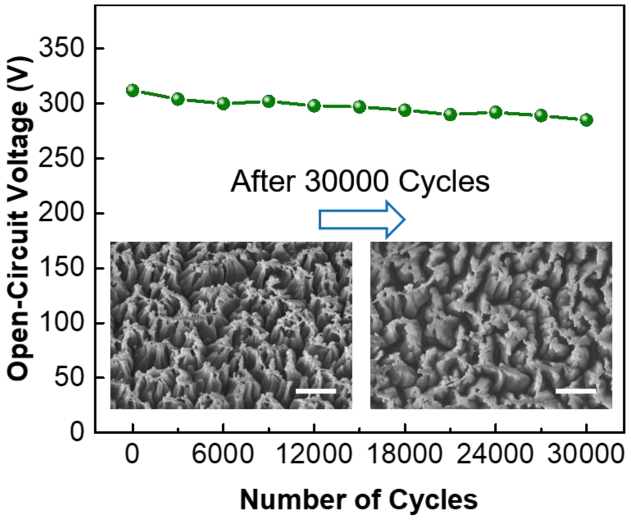

Fig. S3 Robustness and stability investigation of the TENG. Inset: SEM images of the polymer nanowires on PTFE film (scale bar: 1 μm)

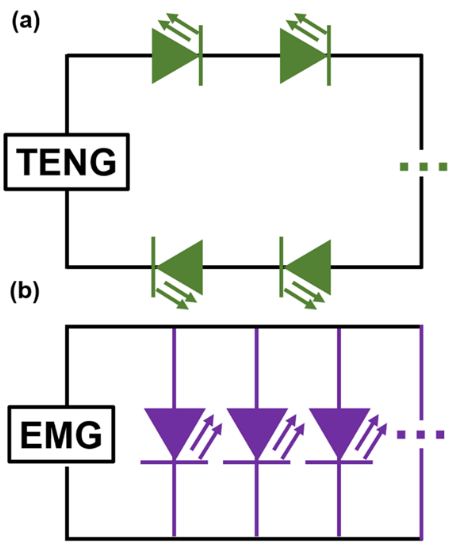

Fig. S4 The circuit diagram of utilizing the device with five CS-TENG units and five RF-EMG units to power electronic devices directly

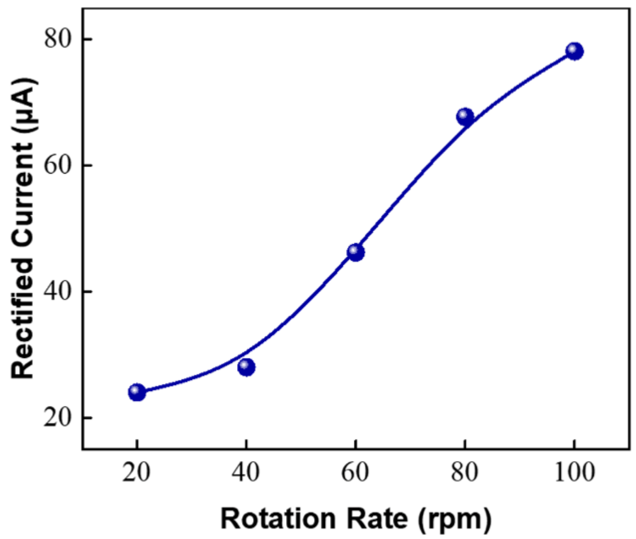

Fig. S5 Dependence of rectified current on rotation rate of CS-TENGs

Table S1 Comparisons of devices for energy harvesting by combining TENG and EMG

Device Structure	Area Volume	TENG *Voc*(V)	TENG *Isc*(μA)	TENG Power	EMG *Voc*(V)	EMG *Isc*(mA)	EMG Power	Ref.
Multilayered WPHG	57 cm^2	500	40	7 mW	1.4	8	4.5 mW	[1]
Rolling TENG	45 cm^3	120	13.5	1.05 μW cm^{-3}	4.92	3.1	1.32 μW cm^{-3}	[2]
Cylindrical TENG	52 cm^2	375	14.12	15.67 μW cm^{-2}	1.79	11.57	27.12 μW cm^{-2}	[3]
Rotary TENG	1508 cm^3	240	7.5	31 μW	2.25	7	11 μW	[4]
Shared-Electrode-Based Hybrid Generator	50 cm^2	250	2.8	0.22 mW	0.13	3.8	0.08 mW	[5]
Fully Enclosed Hybrid Generator	220 cm^3	24	24	0.13 mW	0.8	0.5	0.08 mW	[6]
Hybrid Generator for Air-Flow Energy	60.3 cm^3	/	63.8	3.5 mW	3.7	2.6	1.8 mW	[7]
Hybrid Generator for a watch	38.88 cm^3	/	6.7	0.1 mW	2.1	2.8	6.1 mW	[8]
Multifunctional Power Unit	750 cm^3	142	23.3	31.5 μW	0.66	2.14	66.9 μW	[9]
Triboelectric-Electromagnetic Hybrid Unit	664 cm^3	315.8	44.6	90.7 μW	0.59	1.78	79.6 μW	This work

References

[1] H. Guo, Z. Wen, Y. Zi, M-H. Yeh, J. Wang, L. Zhu, C. Hu, Z.L. Wang, A water-proof triboelectric–electromagnetic hybrid generator for energy harvesting in harsh environments. Adv. Energy Mater. **6**(6), 1501593 (2016). https://doi.org/10.1002/aenm.201501593

[2] X. Wang, Z. Wen, H. Guo, C. Wu, X. He, L. Lin, X. Cao, Z.L. Wang, Fully packaged blue energy harvester by hybridizing a rolling triboelectric nanogenerator and an electromagnetic generator. ACS Nano **10**(12), 11369-11376 (2016). https://doi.org/10.1021/acsnano.6b06622

[3] Z. Wen, H. Guo, Y. Zi, M-H. Yeh, X. Wang et al., Harvesting broad frequency band blue energy by a triboelectric–electromagnetic hybrid nanogenerator. ACS Nano **10**(7), 6526-6534 (2016). https://doi.org/10.1021/acsnano.6b03293

[4] J. Chen, H. Guo, G. Liu, X. Wang, Y. Xi, M.S. Javed, C. Hu, A fully-packaged and robust hybridized generator for harvesting vertical rotation energy in broad frequency band and building up self-powered wireless systems. Nano Energy **33**, 508-514 (2017). http://dx.doi.org/10.1016/j.nanoen.2017.01.052

[5] T. Quan, Z.L. Wang, Y. Yang, A shared-electrode-based hybridized electromagnetic-triboelectric nanogenerator. ACS Appl. Mater. Interfaces **8**(30), 19573-19578 (2016). https://doi.org/10.1021/acsami.6b07162

[6] T. Quan, Y. Yang, Fully enclosed hybrid electromagnetic–triboelectric nanogenerator to scavenge vibrational energy. Nano Res. **9**(8), 2226-2233 (2016). https://doi.org/10.1007/s12274-016-1109-7

[7] X. Wang, S. Wang, Y. Yang, Z.L. Wang, Hybridized electromagnetic–triboelectric nanogenerator for scavenging air-flow energy to sustainably power temperature sensors. ACS Nano **9**(4), 4553-4562 (2015). https://doi.org/10.1021/acsnano.5b01187

[8] T. Quan, X. Wang, Z.L. Wang, Y. Yang, Hybridized electromagnetic–triboelectric nanogenerator for a self-powered electronic watch. ACS Nano **9**(12), 12301-12310 (2015). https://doi.org/10.1021/acsnano.5b05598

[9] H. Shao, Z. Wen, P. Cheng, N. Sun, Q. Shen et al., Multifunctional power unit by hybridizing contact-separate triboelectric nanogenerator, electromagnetic generator and solar cell for harvesting blue energy. Nano Energy **39**, 608-615 (2017). https://doi.org/10.1016/j.nanoen.2017.07.045
